# An Ultrasensitive Electrochemiluminescence Immunoassay for Carbohydrate Antigen 19-9 in Serum Based on Antibody Labeled Fe_3_O_4_ Nanoparticles as Capture Probes and Graphene/CdTe Quantum Dot Bionanoconjugates as Signal Amplifiers

**DOI:** 10.3390/ijms140510397

**Published:** 2013-05-17

**Authors:** Ning Gan, Jing Zhou, Ping Xiong, Tianhua Li, Shan Jiang, Yuting Cao, Qianli Jiang

**Affiliations:** 1 The State Key Laboratory Base of Novel Functional Materials and Preparation Science, School of Material Science and Chemical Engineering, Ningbo University, Ningbo 315211, China; E-Mails: uhghgghh@163.com (J.Z.); xiongping198801@163.com (P.X.); litianhua@nbu.edu.cn (T.L.); caoyuting@nbu.edu.cn (Y.C.); 2 Department of Hematology, Nanfang Hospital, Southern Medical University, Guangzhou 510515, China; E-Mails: jiangsql@yahoo.com.cn (S.J.); jiangqianlid@yahoo.cn (Q.J.)

**Keywords:** dextran-Fe_3_O_4_ magnetic nanoparticles, sandwich immunoassay, graphene CdTe QD nanocomposite, carcinoembryonic antigen 19-9, electrochemiluminescence

## Abstract

The CdTe quantum dots (QDs), graphene nanocomposite (CdTe-G) and dextran–Fe_3_O_4_ magnetic nanoparticles have been synthesized for developing an ultrasensitive electrochemiluminescence (ECL) immunoassay for Carcinoembryonic antigen 19-9 (CA 19-9) in serums. Firstly, the capture probes (CA 19-9 Ab1/Fe_3_O_4_) for enriching CA 19-9 were synthesized by immobilizing the CA 19-9’s first antibody (CA 19-9 Ab1) on magnetic nanoparticles (dextran-Fe_3_O_4_). Secondly, the signal probes (CA 19-9 Ab2/CdTe-G), which can emit an ECL signal, were formed by attaching the secondary CA 19-9 antibody (CA 19-9 Ab2) to the surface of the CdTe-G. Thirdly, the above two probes were used for conjugating with a serial of CA 19-9 concentrations. Graphene can immobilize dozens of CdTe QDs on their surface, which can emit stronger ECL intensity than CdTe QDs. Based on the amplified signal, ultrasensitive antigen detection can be realized. Under the optimal conditions, the ECL signal depended linearly on the logarithm of CA 19-9 concentration from 0.005 to 100 pg/mL, and the detection limit was 0.002 pg/mL. Finally, five samples of human serum were tested, and the results were compared with a time-resolved fluorescence assay (TRFA). The novel immunoassay provides a stable, specific and highly sensitive immunoassay protocol for tumor marker detection at very low levels, which can be applied in early diagnosis of tumor.

## 1. Introduction

Early detection of cancer markers plays a vital role in clinical research, diagnosis and therapy [1]. Carcinoembryonic antigen 19-9 (CA 19-9) is an antigenic determinant associated with many malignant tumors, which is the most widely used tumor marker for the early diagnosis of pancreatic and skin cancers [2]. However, upon the formation of a small tumor, the levels of CA 19-9 rise, so the limits of detection (LODs) of the given methods are important for early screening of tumor [3]. Many immunoassay methods and immunosensors are being developed for detection of CA 19-9, including enzyme-linked immunosorbent assay (ELISA), immunofluorescence, *etc*. [4]. However, most of them are time-consuming, labor-intensive and hazardous to health or require highly qualified personnel and sophisticated instrumentation [5]. In comparison with such above methods, electrochemiluminescence (ECL) immunoassay has a lower background, higher sensitivity, easier operation procedure and simpler instrumentation [6]. ECL has been widely applied in both environmental and biological detection [7]. Many reagents, such as luminol, Tris (2,2′-bipyridyl) ruthenium (II) (Ru(bpy)_3_^2+^), and semiconductor nanocrystals (NPs) have been employed as signal probes to develop highly sensitive selective and simple ECL sensors [8,9]. Among the above signal probes, semiconductor quantum dots (QDs) have attracted wide interests in fabricating ultrasensitive ECL immunosensors based on their advantages of narrow photoemission spectra, high resistance to photo bleaching and broad excitation spectra and, moreover, the freedom in orientation for protein immobilization [10]. Among them, the favorable properties of water-soluble CdTe QDs as a novel biological ECL label over conventional fluorescent probes have attracted considerable interest [11,12]. Searching for a functional nanostructure as the carrier for immobilization of the signal sources materials and antibody has always been the goal in fabricating ultrasensitive ECL immunoassay based on QDs [13]. The emergence of graphene nanosheets provides good opportunities for building highly-sensitive, highly-selective ECL immunosensors, because of its outstanding biocompatibility, good electron transfer, excellent adsorption capacity and large 2-D specific surface area [14,15]. Owing to these excellent properties, the graphene nanosheets is an ideal matrix to coimmobilize CdTe QDs and CA 19-9’s secondary antibody (CA 19-9 Ab2/CdTe-G), which can act as a signal tag to amplify the ECL signal. The hybrid bioconjuncture cannot only provide an interface with very higher surface-to-volume ratio to improve the load density of CdTe QDs on its surface obviously, but also promote electrical communication between CdTe and the sensing surface to amplify the ECL signal to lead to a higher detection sensitivity for target molecules.

Another important procedure for fabricating an ultrasensitive ECL sensor is to synthesize capture probes with a high labeling concentration of antibody; moreover, to be liable to be modified on the surface of the electrode [16]. With the development of nanotechnology, various types of nanomaterials have been widely applied in the fabrication of immunosensor, such as metal nanoparticles [17], graphene (G) [15] and quantum dots (QDs) [14]. In recent years, nano ferromagnetic probes made of nano ferromagnetic oxide (such as Fe_3_O_4_) material have been developed; they can controllably separate under an external magnetic field. This method is convenient, simple, rapid and thorough and has attracted more and more attention of researchers [18]. The magnetic separation technique has widely been applied to various aspects in biotechnology in recent years [19]. In recent years, Fe_3_O_4_ nanoparticles (Fe_3_O_4_ NPs) have been functionalized with many different biological shells for interaction with biological molecules or cells, so as to separate them by a magnetic field [20]. Dextran coated iron oxide nanoparticles (dextran-Fe_3_O_4_) has been widely used in magnetic resonance imaging (MRI) as a contrast enhancement agent for clinical diagnosis [21]. Moreover, it can be used to immobilize an antibody acting as the active targeting probe for enriching an antigen, due to its specific targeting and long half-life in maintaining its bioactivity. Herein, the dextran-Fe_3_O_4_ was synthesized through the co-precipitation method and for labeling the primary antibody of CA 19-9 (CA 19-9 Ab1/dextran-Fe_3_O_4_) as capture probes. The probes cannot only have higher load density for Ab1, because there are so many anchoring sites from –NH_2_ group in its large specific surface area, but also can be easily controlled for enriching CA 19-9 antigens and separated from serum samples with a complex background through an external magnetic field. Screen-printing (thick-film) technology is widely used for the mass-production of disposable electrochemical sensors. Among them, the plane screen printed carbon electrode (SPCE) is widely used because of the lower cost and good conductivity [22]. CA 19-9 Ab1/dextran-Fe_3_O_4_ magnetic probes can be easily immobilized on the surface of SPCE after adding a magnet on the bottom of the plane electrode. After the probes were reacted with CA 19-9 antigen to form the immunocomplex, it can also be liable to be washed away from the electrode after removing the magnet. Thus, a disposable and simply fabricated ECL immune-electrode can be achieved through simple modifying steps by adding or removing a magnetic field.

Up to now, there has been no research using both magnetic capture nanoprobes and graphene/QDs hybrid nanomaterials based signal tag to detect the concentration of protein in human serum and to predict tumor disease. In this work, graphene/CdTe QDs nano bioconjugates and dextran-Fe_3_O_4_ magnetic nanoparticles were both used in detecting the concentration of CA 19-9. The novel magnetic nanoparticles were firstly prepared and conjugated with mouse anti-human CA 19-9 antibody (Ab1) as capture probes. Then, graphene/CdTe QDs bionanoconjugates were synthesized and conjugated with CA 19-9’s secondary antibody (Ab2). The sandwich immunoreaction occurred. After the reaction, the superfluous CA 19-9 Ab2/CdTe-G complexes were washed away. The relationship between the ECL intensity of the complex and the concentration of CA 19-9 was determined. Finally, several patients’ samples of human serum were tested based on the immunoassay.

## 2. Results and Discussion

### 2.1. Spectral Characterization of the CdTe-G and Dextran-Fe_3_O_4_ Nanocomposite

**Figure 1:**
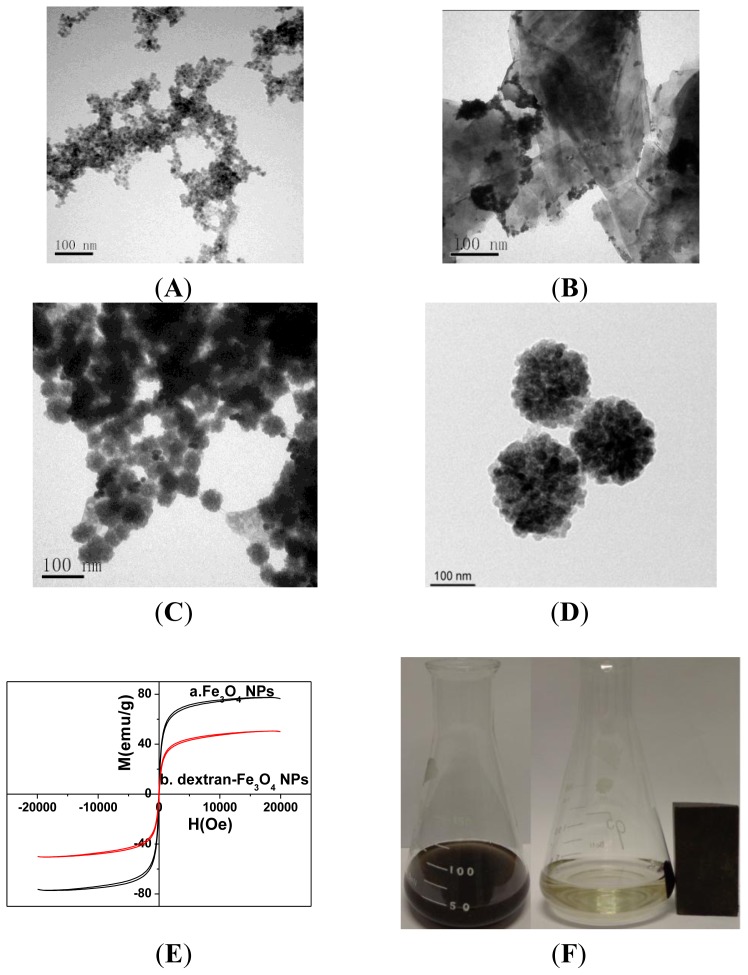
TEM image of the CdTe QDs (**A**); CdTe-G nanosheets (**B**); Fe_3_O_4_ NPs (**C**) and dextran-Fe_3_O_4_ NPs (**D**); Magnetization hysteresis for (**a**) Fe_3_O_4_ and (**b**) dextran-Fe_3_O_4_ nanoparticles recorded at room temperature (**E**); Dextran-Fe_3_O_4_ NPs in the absence (**right**) and presence (**left**) of an external magnetic field (**F**).

Figure 1 shows the property characterization of the CdTe-G and dextran-Fe_3_O_4_ by transmission electron microscope (TEM). Figure 1A shows the typical image of CdTe QDs, whose diameter was about 5 nm. The ODs were evenly dispersed on graphene (Figure 1B). In the fluorescence images of graphene, QD were obtained. The absorption peak of CdTe QDs was at 520 nm, while the photoluminescence peak was at 560 nm. The narrow peak indicated the small size distribution of the as-prepared CdTe QDs. Dissolved graphene alone yielded no fluorescence, while CdTe QDs were dispersed onto the surface of graphene sheets. The pictures of CdTe-G complex shows a strong emission waveform at 560 nm, which revealed successful attachment of fluorescent QD to graphenes.

The TEM image of Fe_3_O_4_ NPs (Figure 1C) shows that its diameter was about 80 nm and dextran-Fe_3_O_4_ NPs (Figure 1D) about 200 nm, which means that dextran was closely and evenly covered on the surface of Fe_3_O_4_ NPs. The vibration sample magnetometer (VSM) magnetization curves of Fe_3_O_4_ NPs and dextran-Fe_3_O_4_ NPs were recorded in Figure 1E at room temperature (298 K). It is evident that the sample presented a saturation magnetization (Ms) of 74.3 emu g^−1^ and 47.2 emu g^−1^ for Fe_3_O_4_ NPs and dextran-Fe_3_O_4_ NPs. It can be shown that the saturation magnetization of dextran-Fe_3_O_4_ NPs reduced after coating with a layer of dextran. The magnetic coercive force of Fe_3_O_4_ and dextran-Fe_3_O_4_ NPs was, respectively, 10.2 and 14.3 O_e_, which means that the above two particles show good paramagnetism, but not superparamagnetism features. In Figure 1F, fextran-Fe_3_O_4_ could be homogeneously distributed in the solution without the magnet. Once an external magnetic field is applied, it can be attracted quickly towards the magnet, leaving the bulk solution clear and transparent. Once the magnet was removed, the probe dispersed evenly in the solution again, which indicated its good paramagnetic features.

### 2.2. ECL Characterization of CdTe, CdTe-G and CA 19-9 Ab2/CdTe-G Signal Tag

**Figure 2:**
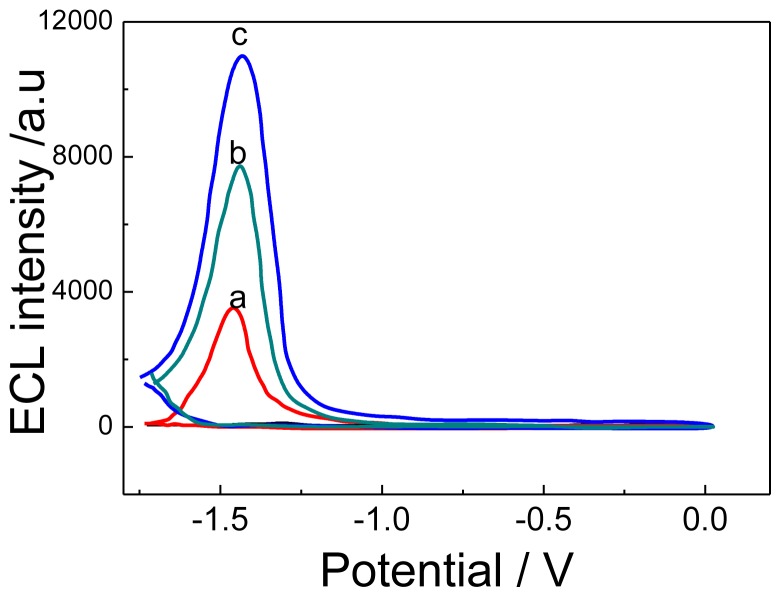
Electrochemiluminescence (ECL)-potential curves of CdTe QDs (**a**); CdTe-G nanosheets (**b**) and CA 19-9 Ab2/CdTe-G bioconjuncture (**c**) modified SPCE electrode. The voltage of the photomultiplier tube was set at 600 V.

Figure 2a showed the ECL-potential curve of CdTe QDs, which could generate weak ECL, but the ECL intensity of CdTe-G composite film (curve b) on SPCE was much higher than that of CdTe QDs (curve b), suggesting that graphene nanosheets have better electric conductivity and a more specific surface area to facilitate the ECL reaction, which was more favorable for fabricating an ultrasensitive ECL immunosensor. Figure 2c shows that the CA 19-9 Ab2/CdTe-G bioconjuncture can also emit an ECL signal, while its intensity was lower than CdTe-G, which suggested that CA 19-9 Ab2 did not emit an ECL signal and can hinder the electron transfer between CdTe-G and the SPCE electrode. Furthermore, the CA 19-9 Ab2/CdTe-G bioconjuncture can act as signal tag for detecting the target antigen.

### 2.3. Characterization of the ECL Immunosensor

#### 2.3.1. ECL Behavior

**Figure 3:**
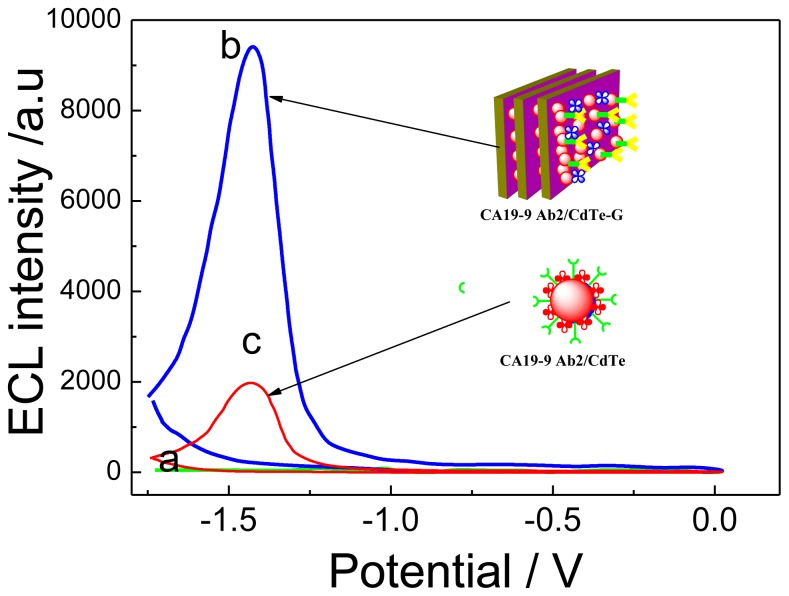
ECL-potential curves of the CA 19-9 Ab1/dextran-Fe_3_O_4_ modified SPCE electrode (**a**); (a) incubated in 10 pg/mL CA 19-9 + CA 19-9 Ab2/CdTe-G (**b**); (a) incubated in 10 pg/mL CA 19-9 + CA 19-9 Ab2/CdTe in 0.1 mol/L PBS (pH 7.0) containing 0.1 mol/L KCl and 0.1 mol/L K_2_S_2_O_8_ (**c**). The voltage of the photomultiplier tube was set at 600 V.

The fabrication process of the ECL immunosensor was monitored by measuring the ECL signals after each immobilization step (Figure 3). It could be seen that there was no ECL signal for capture probes (CA 19-9 Ab1/dextran-Fe_3_O_4_) (curve a); whereas, there was an obvious enhancement of ECL intensity after a sandwich immunoreaction of primary antibody on the magnetic nanoparticles (CA 19-9 Ab1/dextran-Fe_3_O_4_), 10 pg/mL CA 19-9 or serum sample (curve b) and the secondary antibody labeled with CdTe-G nanocomposite. Five-fold enhancement in the ECL signal for CA 19-9 (10 pg/mL) detection by the above CA 19-9 Ab1/dextran-Fe_3_O_4_ tag were achieved when comparing with CdTe as signal tag matrix (CA 19-9 Ab2/CdTe) (curve c). All these imply that the graphene can greatly accelerate the electron transfer speed between CdTe QDs and the SPCE electrode and, then, obviously amplify the ECL signal.

#### 2.3.2. Electrochemical Impedance (EIS) Behavior

**Figure 4:**
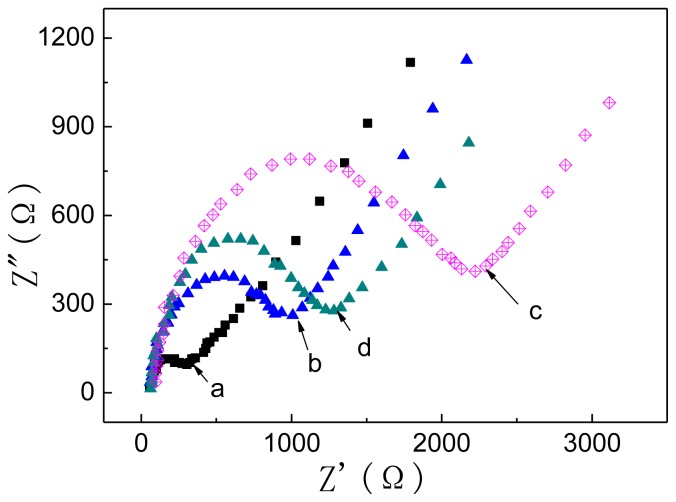
Electrochemical impedance of screen printed carbon electrode (SPCE) (**a**); CA 19-9 Ab1/dextran-Fe_3_O_4_ modified SPCE (**b**); (b) + 10 pg/mL CA 19-9 (**c**); (c) + CA 19-9 Ab2/CdTe-G (**d**) in 0.1 mol/L PBS [containing 2 mmol/L Fe(CN)_6_^4−/3−^ and 0.1 mol/L KCl, pH 7.0]. The frequency range is between 0.01 and 100,000 Hz with a signal amplitude of 5 mV.

Electrochemical impedance (EIS) is an effective method for monitoring the changes in the surface features of the electro-immunosensor in the assembly process, and it is widely used to characterize the fabrication process of the ECL immunosensor [23]. Figure 4 shows the EIS of the electrode during the stepwise modification processes in 0.1 mol/L PBS containing 2 mmol/L Fe(CN)_6_]^3−/4−^ and 0.1 mol/L KCl. Results revealed that CA 19-9 Ab1/dextran-Fe_3_O_4_ modified electrode (curve a) shows a higher electron transfer resistance, *R*_et_(about 350 Ω), than the bare SPCE one (curve b), which can be explained by CA 19-9 Ab1 and dextran both being nonconductive components. After the nonconductive CA 19-9 is incubated to the capture probes on the electrode (curve c), *R*_et_ increased to about 1200 Ω. Subsequently, when the signal tag (CA 19-9 Ab2/CdTe-G) was further incubated onto the immune-electrode to form the sandwich complex between the capture probe and the CA 19-9 antigen, the resistance for the redox probe obviously decreased (curve d) due to the excellent electrical conductivity of CdTe-G.

#### 2.3.3. Optimization of Experimental Conditions

**Figure 5:**
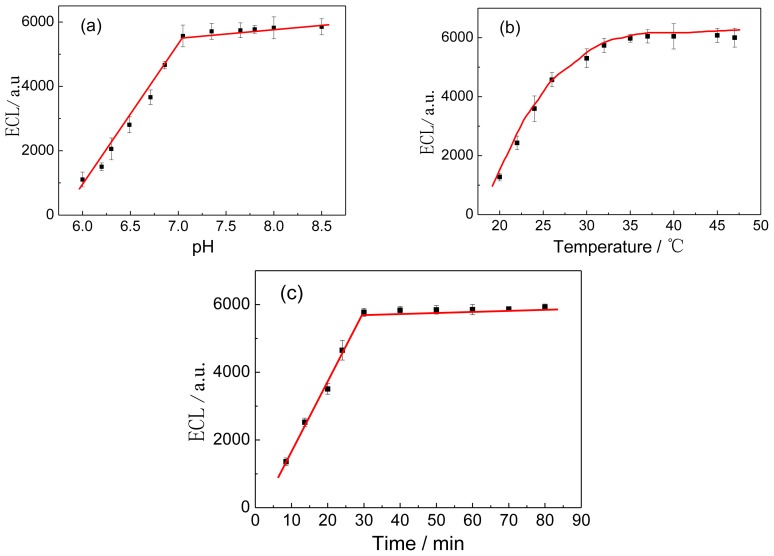
Effect of pH (**a**); incubation temperature (**b**) and incubation time (**c**) on the quenched ECL intensity of the immunosensor toward 5 pg/mL CA 19-9. The voltage of the photomultiplier tube was set at 600 V.

Figure 5 shows the immunoassay conditions, such as the amount of the CA 19-9 Ab1/dextran-Fe_3_O_4_ composite solutions absorbed on the electrode, pH of the supporting electrolyte, incubation temperature and incubation time, which can affect the ECL response of the immunosensor.

The concentration of CA 19-9 Ab1/dextran-Fe_3_O_4_ capture probes can highly influence the performance of the ECL signal response. The concentration of the probes solution (0.2, 0.3, 0.5, 1, 1.5, 3.0, 4.0 and 5.0 mg mL^−1^) was chosen for experiments. Results revealed that 2 mg/mL CA 19-9 Ab1/dextran-Fe_3_O_4_ solution was optimal. Further, based on the experimental results, 10 μL of capture probes’ solution was selected as the optimal amount dropped on the electrode surface, which could be easily absorbed on the surface of the crystal electrode by magnet.

The pH of the background solution could greatly affect the ECL response of the immunosensor, because the activity of the antibody protein might be influenced by the acidity of the solution. Thus, the effect of pH from 6.0 to 8.5 on the immunosensor performance was investigated using 5 pg/mL CA 19-9 solutions. As shown in Figure 5a, the maximum ECL intensity could be obtained at pH 7.0 by the immunosensor. Thus, the detection was performed in pH 7.0 PBS throughout the experiment.

The effect of incubation temperature and time on the ECL response of the immunosensor was also investigated. It could be seen from Figure 5b that the ECL signal first increased and then reduced with the increase of incubation temperature from 20 to 50 °C, and a maximum Δ*I*_ECL_ was obtained at 37 °C. Figure 5c shows that ECL signal increased with the increase of incubation time and reached a plateau at 30 min. Therefore, 37 °C and 30 min were selected as the optimum incubation temperature and time in this study.

### 2.4. Analytical Performance

**Figure 6:**
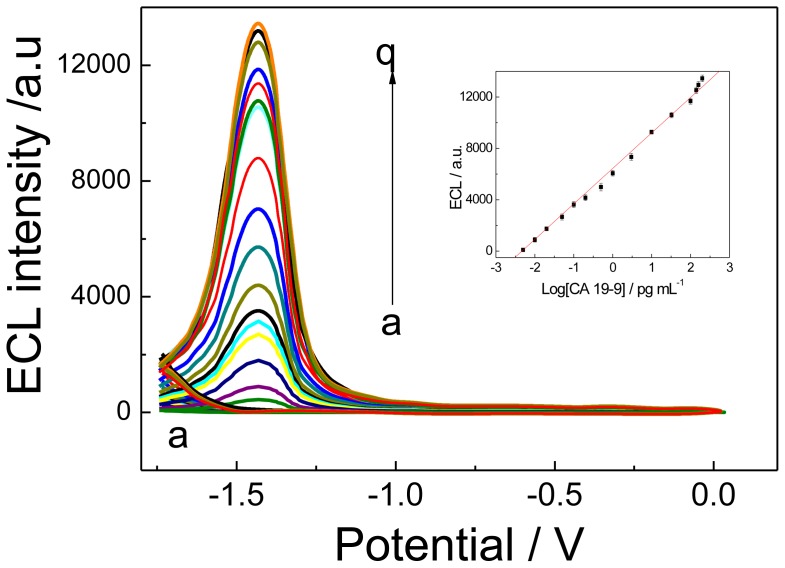
ECL profiles of the immunosensor in the absence (**a**) and presence (**b**→**h**) of different concentrations of CA 19-9 in 0.1 mol/L PBS (pH 7.0) containing 0.1 mol/L KCl and 0.1 mol/L K_2_S_2_O_8_. CA 19-9 concentration (pg/mL): 0 (a), 0.005 (b), 0.01 (c), 0.02 (d), 0.05 (e), 0.1 (f), 0.2 (g), 0.5 (h), 1 (i), 3 (j), 10 (k), 30 (l), 100 (m), 140 (n), 150 (o), 160 (p), 200 (q). The voltage of the photomultiplier tube was set at 700 V. Scan rate: 100 mV/s. The inset: calibration curve for Ag determination.

Under the optimal conditions, the ECL immunosensor was carried out to analyze various concentrations of CA 19-9 standard solution (Figure 6), and the ECL response was recorded. The intensity of the ECL response (*I*) increased with the increase of CA 19-9 concentration. The linear regression equation was *I* = 6356.3 + 2820 × log (CA 19-9), with a coefficient of 0.9962. The detection range was from 0.005 to 100 pg/mL, and the limit of detection was 0.002 pg/mL. According to the linear equation, we could detect Ag concentration quantitatively. Higher CA 19-9 levels could be detected by an appropriate dilution with PBS. Due to signal amplification from the high loading of CdTe QDs, a 10^6^-fold enhancement in ECL signals for CA 19-9 detection was achieved compared to the ELISA method [24].

### 2.5. Specificity, Reproducibility and Stability of the ECL Immunosensor

To investigate the specificity of the fabricated immunosensor, an assay was performed in a 5 pg/mL CA 19-9 sample solution containing the interfering substances of 2 ng/mL alpha fetoprotein (AFP), 10 ng/mL human chorionic gonadotropin (HCG) and 50 ng/mL human IgG. The mixed sample solution was measured by the immunosensor, and the results were compared with that of the standard CA 19-9 solution (100 pg/mL). The ELC intensity variation due to the interfering substances was less than 2.3% of that without interferences, indicating the selectivity of the immunosensor was satisfactory.

The reproducibility of the immunosensor was evaluated by detecting 5 pg/mL CA 19-9 by the same SPCE electrode. The relative standard deviation (RSD) of the measurements for the five immunosensors was 3.5%, indicating the excellent precision and reproducibility of the immunosensor.

Regeneration of the immunosensor was examined by detecting 5 pg/mL CA 19-9 with the renewed immunosensor. The immunosensor was regenerated by removing the added magnet at the back of the SPCE, then washing with pH 8.5 boric acid buffer solution for 2 min to remove the magnetic immunocomplex. The consecutive measurements were repeated 20 times; an average recovery of 95.4% and an intra-assay RSD of 2.3% were acquired. The results demonstrated that the proposed immunosensor could be regenerated simply by removing the exerted magnetic field.

After the immunosensor was stored at 4 °C over two weeks, it was used to detect the same CA 19-9 concentration. The response of the immunosensor retained about 95% of its initial value, demonstrating that the immunosensor had a good stability. Thus, the developed immunosensor is an appropriate tool for the detection of CA 19-9 based on the obtained results.

### 2.6. Application of the Immunosensor in Serum Samples

Direct determination of CA 19-9 level in the blank human serum (pretreated to make them without CA 19-9) is very important in the early detection of oral cancer. In order to investigate the applicability and reliability of the prepared ECL immunosensor for clinical applications, recovery experiments were performed by standard addition methods in human serum and saliva samples. Results, as listed in Table 1, showed an acceptable recovery in the range of 90%–101%. The results were consistent with that by time-resolved fluorescence assay (TRFA), which also revealed that the developed ECL immunosensor may provide an efficient tool for sensitive determination of the ultratrace level of CA 19-9 in human serum, almost as well as TRFA, while its instrument and procedures are relatively simple.

## 3. Experimental Section

### 3.1. Reagents and Chemicals

Graphite powder (8000 mesh was purchased from Aladdin Co, Ltd, Shanghai, China), potassium permanganate, hydrochloric acid and sodium hydroxide, hydrazine monohydrate, hydrogen peroxide, ammonia solution, sodium borohydride, (NH_4_)_2_Fe(SO_4_)_2_·6H_2_O, NH_4_Fe(SO_4_)_2_·12H_2_O, CdCl_2_ and NaHTe were obtained from Sinopharm Chemical Reagent Co., Ltd. (Shanghai, China). Experimental agents, 3-dimethylaminopropyl-*N*-ethylcarbodiimide hydrochloride (EDC) (99%), H_2_SO_4_ (95%), HNO_3_ (70%), potassium peroxydisulfate (K_2_S_2_O_8_, 99 wt.%), 4% paraformaldehyde, *N*-hydroxysuccinimide (NHS), phosphate buffered saline (PBS), bovine serum albumin (BSA), fetal calf serum (FCS) and monoclonal anti-CA 19-9 antibody, were purchased from Abcam^®^ (Cambridge, UK). Octa-ammonium polyhedral oligomeric silsesquioxanes (Octa-ammonium-POSS) was purchased from Hybrid Plastics^®^ (Hattiesburg, MS, USA). All chemicals were of analytical grade.

### 3.2. Apparatus

Electrochemical experiments were performed on an electrochemical workstation CHI -660C (Shanghai Chenhua Instrument Co., Shanghai, China). SPCE were purchased from eDAQ Technology Co. (Madrid, Spain). A three compartment electrochemical cell contained a saturated calomel reference electrode, a platinum wire auxiliary electrode and the modified SPCE (Φ = 3.0 mm) as the working electrode. The electrochemical impedance experiment was carried out in 5 mmol/L Fe(CN)_6_^4−/3−^ containing 0.1 mol/L KCl with frequencies ranging from 100 kHz to 0.1 Hz with an amplitude of 5 mV. The cyclic voltammetry (CV) was performed in 0.1 mol/L pH 7.0 PBS containing 0.1 mol/L KCl. The measurements were carried out at room temperature (25 ± 0.5 °C).

### 3.3. Synthesis of Dextran-Fe_3_O_4_ Magnetic Nanoparticles and CdTe-G

#### 3.3.1. Dextran-Fe_3_O_4_ Magnetic Nanoparticles

Firstly, nano-Fe_3_O_4_ particles were synthesized by the method of coprecipitation according to the previous protocol [25]. Five grams of FeSO_4_·7H_2_O and 3 g FeCl_3_·6H_2_O were dissolved in 100.0 mL of water, and the solution was deaerated under a robust flow of nitrogen with stirring at room temperature for 30 min. Then the solution was sonicated for 10 min, 2 mL 1 g/mL dextran. 8 mL of 8 mol L^−1^ NH_4_OH aqueous solution was added dropwise to precipitate the iron oxides, while the mixture solution was sonicated. To promote the complete growth of the nanoparticle crystals, the reaction was carried out at 60 °C for 60 min under constant mechanical stirring. To purify the magnetic nanoparticles, the unbound dextran and other impurities were washed away in a high-gradient magnetic field 5 times. The precipitate was isolated in the magnetic field, and the supernatant was separated from the precipitate by decantation. The obtained Fe_3_O_4_ was then washed with absolute alcohol three times and dried under vacuum. Finally, the nanoparticles were dispersed in 10.0 mL of ultra-pure water.

#### 3.3.2. CdTe-G

Firstly, amino-graphene (G-NH_2_) was synthesized according to literature [26]. Three milligrams of graphenes were mixed with 1.5 mg EDC and 0.9 mg NHS, dissolved in 3 mL of DMF and stirred at room temperature for 2 h. Then, it was followed by ultrasonication for 3 min at room temperature to improve graphenes dispersion. Three milligrams of Octa-Ammonium-POSS was dissolved in 1 mL of 1 mol/L NaOH. Graphenes and Octa-Ammonium-POSS solutions were mixed together and stirred at room temperature for 1 h. The mixture was then taken and centrifuged for 15 min at 5000 rpm. Distilled water was added to the precipitate and the centrifuge cycle repeated. The final precipitate was oven dried for 10 min at 65 °C and dried weight calculated. PBS was then added to obtain 1 mg/mL concentration.

The water-soluble CdTe QDs were prepared by using the reaction between Cd^2+^ and NaHTe in the presence of l-Cysteine (l-Cys) as the stabilizing agent following the method described by Zhang *et al*. [27]. CdS dots (QDs) with free surface –COOH groups and an emission spectrum of 600 nm were used. CdS QDs solution was mixed with methanol at 1:1 volume ratio and centrifuged at 5000 rpm at room temperature for 10 min. The supernatant was then discarded and PBS added to the weighed precipitate to obtain 1 mg/mL concentration. QDs and G-NH_2_ solutions were combined to make up a final solution of 1:1 concentration ratio. Then, the graphen-NH_2_-CdTe QDs (CdTe-G) complex was obtained.

### 3.4. Immobilization of CA 19-9 Secondary Antibodies to CdTe-G (CA 19-9 Ab2/CdTe-G)

A 10 μL anti-CA 19-9 antibody concentrate was diluted into one container with 1 mL of antibody Diluent Dako REAL™ (Dako, Glostrup, UK). To 1 mL of CdTe-G complex, 5 mg of NHS and 5 mg EDC were added and placed on a shaker for 20 min at room temperature. One hundred microliters of the diluted antibody solution were added to the CdTe-G complex and placed on a shaker for 10 min. The mixtures were then centrifuged using a 100 kDa filter system at room temperature for 10 min at 5000 rpm, the supernatant discarded, PBS added to the pre-marked level and mixed. The solution was further coated in the mixture of 10 mg/mL calf thymus DNA after gently stirring in a magnetic file for 6 h. Then, the solution of CA 19-9 Ab2/CdTe-G signal probes were obtained and then evaluated by transmission electron microscopy images (TEM).

### 3.5. Immobilization of the Primary Antibody of CA 19-9 on the Surface of the Magnetic Dextran-Fe_3_O_4_ Nanoparticles (CA 19-9 Ab1/Dextran- Fe_3_O_4_)

**Scheme 1:**
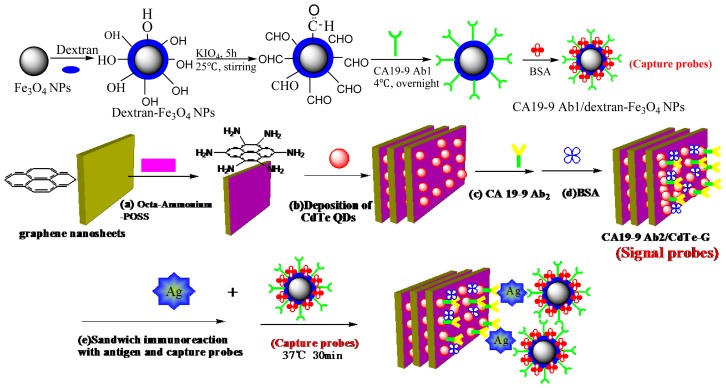
Schematic processes of the immunosensor fabrication process and detection principle. Dropping of Nafion membrane (**a**); electrochemical deposition of HAuCl_4_ (**b**); and immobilization of CA19-9 Ab_2_ (**c**) and blocked with BSA (**d**); immunoreaction of CA 19-9 and capture probes (**e**), resulting in an amplified signal-generating detection.

2 mL of 5 mg/mL dextran-Fe_3_O_4_ magnetic nanoparticles and 2.0 mL of 1.0 × 10^−2^ mol/L KIO_4_ were mixed and then aerated with N_2_ for another 20 min. Then, the mixture was stirred for 5 h at 10 °C. After the reaction, the precipitate was washed by ultra-pure water 5 times. The precipitates were dispersed in pH 8.5 borate buffer solution, and the final volume was 10 mL. One hundred microliters of CA 19-9 primary antibody were added to the mixture, and the resultant was placed at 4 °C overnight. One millimeter of 1.0% bovine serum albumin (BSA) was added, and the mixture was placed at 4 °C overnight again. Preparation of dextran-Fe_3_O_4_ and immobilization of the primary antibody (CA 19-9 Ab1/dextran-Fe_3_O_4_) on the surface of magnetic nanoparticles are shown in Scheme 1.

### 3.6. ECL Immunoassay for CA 19-9

**Scheme 2:**
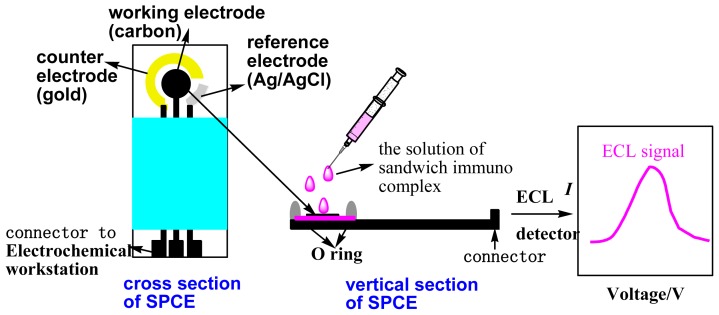
The immunoassay procedure [28].

A schematic representation of the steps used to perform the electrochemical ELISA was shown in Scheme 2  [28]. Different amounts of human serums were introduced into the tubes containing 300 μL of the 2 mg/mL CA 19-9 Ab1/dextran-Fe_3_O_4_. The mixture was incubated for 30 min at room temperature. When the reaction was over and the CA 19-9/CA 19-9 Ab1/dextran-Fe_3_O_4_ conjugation was formed, 200 μL of the CA 19-9 Ab2/CdTe–G was added to the above tube and incubated for another 30 min at room temperature, and then the incubation sandwich-type complex was formed. The mixture was magnetically purified for 5 min, and the precipitate was washed twice in a high-gradient magnetic field using pH 7.0 PBS buffer solution. Finally, the sandwich-type complex was dispersed in 1.0 mL of pH 7.0 PBS. The solution was added dropwise to the electrode’s surface, whose back was placed with a magnet in advance. The electrodes were scanned from 0 to −1.6 V with a scan rate of 100 mV/s, and then ECL signals related to the CA 19-9 concentrations were measured. After each detection, the electrode was instantaneously washed with PBS solution 5 times to remove the precipitation solution. Because the magnetic precipitation was dropped on the electrode, it can be easily washed away after removing the magnet; thus, the immunosensor can be renewed.

## 4. Conclusions

In summary, we demonstrated an ultrasensitive ECL-sensing protocol for detecting CA 19-9 using a sandwich immunoreaction strategy based on novel magnetic capture probes and a graphene/quantum dots labeled signal tag. The immunoassay has several distinct advantages, such as following: (1) the usage of CdTe/graphene nanocomposite as the signal tag can display good biocompatibility and greatly enhanced ECL intensity. The CdTe/graphene label was compared with the only CdTe (FITC) label. The previous is five-folds of magnitude more sensitive than the latter; (2) The magnetic capture probe by dextran-Fe_3_O_4_ can be used for separation and enrichment of the ultratrace level of CA 19-9 antigen in serum; (3) After detection, the sandwich immunocomplex can be easily moved from the surfaces of the electrodes by removing the external magnetic field, so as to make the immunosensor renewable; (4) The ECL immunoassay was ultrasensitive enough for detecting a tiny concentration change for tumor markers at the level of pg/mL. Its detection results in real human serum samples are in good agreement with those obtained by TRFA, while its instrument and procedures are rather simpler. This technique may be applied in many types of antibody–antigen systems in clinical practice.

## Figures and Tables

**Table 1:** Recovery tests for CA 19-9 in spiked human serum and saliva samples (*χ̄* ± *s*, pg/mL, *n* = 3).

Sample	Added (pg/mL)	Found by the method	Recovery (%)	Time-resolved fluorescence assay, TRFA
Serum 1	0.002	0.0022 ± 0.0001	101	0.002
Serum 2	0.02	0.017 ± 0.003	98.5	0.019
Serum 3	0.2	0.22 ± 0.01	100	0.22
Serum 4	2	1.82 ± 0.02	90.0	1.93
Serum 5	20	19.6 ± 0.7	98.0	20.4
